# Development and evaluation of a non-ribosomal random PCR and next-generation sequencing based assay for detection and sequencing of hand, foot and mouth disease pathogens

**DOI:** 10.1186/s12985-016-0580-9

**Published:** 2016-07-07

**Authors:** Anh To Nguyen, Thanh Tan Tran, Van Minh Tu Hoang, Ngoc My Nghiem, Nhu Nguyen Truc Le, Thanh Thi My Le, Qui Tu Phan, Khanh Huu Truong, Nhan Nguyen Thanh Le, Viet Lu Ho, Viet Chau Do, Tuan Manh Ha, Hung Thanh Nguyen, Chau Van Vinh Nguyen, Guy Thwaites, H. Rogier van Doorn, Tan Van Le

**Affiliations:** Oxford University Clinical Research Unit, 764 Vo Van Kiet Street, Ward 1, District 5, Ho Chi Minh City, Vietnam; Children’s Hospital 2, Ho Chi Minh City, Vietnam; Hospital for Tropical Diseases, Ho Chi Minh City, Vietnam; Children’s Hospital 1, Ho Chi Minh City, Vietnam; Centre for Tropical Medicine, Nuffield Department of Medicine, University of Oxford, Oxford, UK

**Keywords:** Hand, foot and mouth disease, Enterovirus A, Random PCR, FR26RV-Endoh primer, Next-generation sequencing

## Abstract

**Background:**

Hand, foot and mouth disease (HFMD) has become a major public health problem across the Asia-Pacific region, and is commonly caused by enterovirus A71 (EV-A71) and coxsackievirus A6 (CV-A6), CV-A10 and CV-A16. Generating pathogen whole-genome sequences is essential for understanding their evolutionary biology. The frequent replacements among EV serotypes and a limited numbers of available whole-genome sequences hinder the development of overlapping PCRs for whole-genome sequencing.

We developed and evaluated a non-ribosomal random PCR (rPCR) and next-generation sequencing based assay for sequence-independent whole-genome amplification and sequencing of HFMD pathogens. A total of 16 EV-A71/CV-A6/CV-A10/CV-A16 PCR positive rectal/throat swabs (Cp values: 20.9–33.3) were used for assay evaluation.

**Results:**

Our assay evidently outperformed the conventional rPCR in terms of the total number of EV-A71 reads and the percentage of EV-A71 reads: 2.6 % (1275/50,000 reads) vs. 0.1 % (31/50,000) and 6 % (3008/50,000) vs. 0.9 % (433/50,000) for two samples with Cp values of 30 and 26, respectively. Additionally the assay could generate genome sequences with the percentages of coverage of 94–100 % of 4 different enterovirus serotypes in 73 % of the tested samples, representing the first whole-genome sequences of CV-A6/10/16 from Vietnam, and could assign correctly serotyping results in 100 % of 24 tested specimens. In all but three the obtained consensuses of two replicates from the same sample were 100 % identical, suggesting that our assay is highly reproducible.

**Conclusions:**

In conclusion, we have successfully developed a non-ribosomal rPCR and next-generation sequencing based assay for sensitive detection and direct whole-genome sequencing of HFMD pathogens from clinical samples.

**Electronic supplementary material:**

The online version of this article (doi:10.1186/s12985-016-0580-9) contains supplementary material, which is available to authorized users.

## Background

Hand, foot and mouth disease (HFMD) is a common and usually mild disease of children worldwide. The disease is caused by different genotypes of the species Enterovirus A, genus *Enterovirus*, family *Picornaviridae* (including coxsackievirus A (CV-A) 6, 10 and 16 and particularly EV-A71). However, EV-A71 has emerged and caused large and sometimes severe/fatal HFMD outbreaks [1] across the Asia-Pacific region since 1997. Of note, the frequent replacements between EV-As have been observed over the last decade in the regions where HFMD is endemic [2–6]. In recent years CV-A6 has emerged and replaced CV-A16 to become the dominant EV-A detected in HFMD patients [7, 8]. While the underlying mechanism of this phenomenon remains unknown, the data highlight the importance of continued effort to monitor the evolution of the causative agents of HFMD.

Currently, there is no clinically proven antiviral drug available to treat severe disease. Likewise, although phase III trials of three monovalent inactivated EV-A71 vaccines have been completed in China with an efficacy of over 95 %, routine use is still far away. Moreover, to what degree the implementation of a monovalent vaccine for EV-A71 may influence the epidemic patterns of HFMD and the evolution of the causative agents in endemic countries is a subject that merits follow-up research.

Collectively, the ability to generate viral whole-genome sequences is essential for understanding the evolutionary biology and epidemiology of HFMD. It is also important for the development of intervention strategies, especially vaccines. While the availability of relatively large numbers of EV-A71 whole-genome sequences (*n* = ~524) deposited in GenBank has facilitated the development of a sensitive overlapping PCR based whole-genome sequencing assay [9], smaller numbers of whole-genome sequences of other EV-As are available (CV-A16; *n* = 61, A6; 35, A10; 11) from limited localities. This is problematic for the selection of specific PCR primers that can amplify diverse EV-As. Additionally, one of the major drawbacks of specific-PCR based sequencing assays is that due to the nature of quick evolution rates of RNA viruses, selected primers may need to be adjusted regularly to be able to amplify newly emerging viral variants or genotypes. As a consequence, a sequence-independent approach is thus attractive to overcome such obstacles.

Developed by Froussard in 1992 [10], random PCR (rPCR) primer (FR26RV-N6: 5′-GCCGGAGCTCTGCAGATATCNNNNNN-3′) consists of a fixed 20 nucleotides (FR20RV: GCCGGAGCTCTGCAGATATC) at the 5′-end and a random hexanucleotides at 3′ end (N6: NNNNNN). In 2005 Endoh and his colleagues designed a set of 96 hexanucleotides for specific amplification of viral sequences called non-ribosomal hexanucleotides [11]. For sequence-independent whole-genome amplification and sequencing of HFMD pathogens, herein we describe the development and evaluation of a non-ribosomal random amplification assay utilizing the 96 non-ribosomal hexanucleotide oligos designed by Endoh [11] and the 5′-end fixed oligo of the conventional random PCR primers (FR20RV) [10]. When combined with next-generation sequencing, our assay showed that it could generate full-genome sequences of HFMD pathogens directly from clinical specimens.

## Methods

### Samples

The clinical samples used included two residual throat swabs from anonymous HFMD patients with EV-A71 infection admitted to the Hospital for Tropical Diseases in Ho Chi Minh City in 2012. Additionally, 13 throat/rectal swabs of diverse viral load (including CV-A6; *n* = 4, CV-A10; *n* = 4, CV-A16; *n* = 3 and EV-A71; *n* = 2) derived from patients enrolled into an on-going prospective observational HFMD study of all severities in three referral hospitals in Ho Chi Minh City, Vietnam since 2013 were also used [9]. The clinical samples were collected in viral transport medium, divided into three aliquots and stored at -80 °C until use. Viral detection and serotype identification were done as per the study protocol using previous described assays [12, 13].

### Development and preparation of non-ribosomal random PCR primers

For selective amplification of viral sequences, we replaced the random hexanucleotide motif at the 3′-end of the primer FR26RV-N6 by those 96 hexanucleotides designed by Endoh. This resulted in a set of 96 separate primers consisting of an FR20RV sequence at 5′-end plus one of the 96 Endoh’s hexanucleotides at the 3′-end (Additional file 1: Table S1).

Each individual primer was synthesized at a concentration of 100 μM, and an equal amount of each synthesized oligo was pooled together to make working solution (~1 μM). This primer mixture was named FR26RV-Endoh.

### Sample pretreatment and nucleic acid extraction

**Fig. 1:**
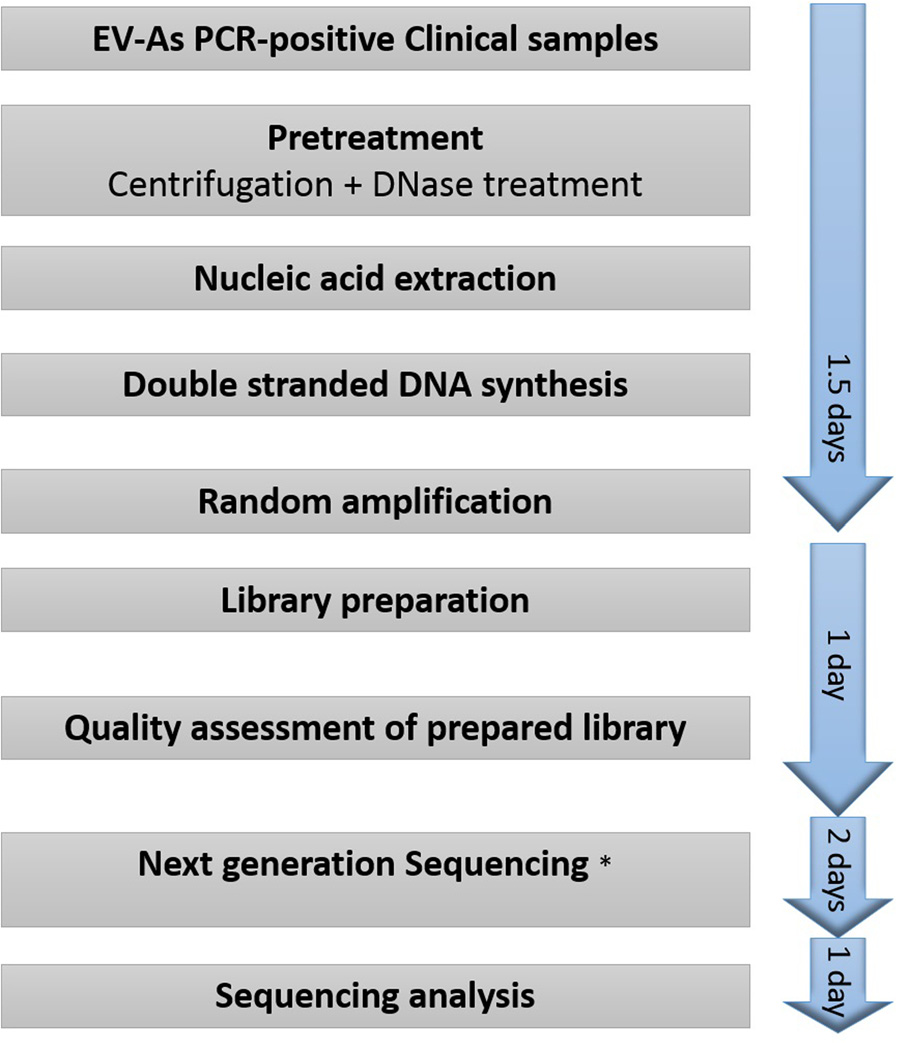
Flowchart showing an overview of the whole procedure of rPCR-Miseq based assay. Note: * the turn-around time may vary, especially when using service platform, which may take more than 2 days

An overview of the whole procedure is described in Fig. 1. Sample pretreatment was carried out as previously described [14]. In short, prior to nucleic acid isolation 110 μl of clinical samples was centrifuged at 10,000 g for 10 min. The resulting 100 μl of supernatants were collected and treated with 2U/ul of turbo DNase (Ambion, Life Technology, Carlsbad, CA, USA) at 37 °C for 30 min. Viral RNA was then extracted from the treated material using QIAamp viral RNA kit (QIAgen GmbH, Hilden, Germany), following the manufacturer’s instructions, and finally eluted in 50 μl of elution buffer (provided with the extraction kit).

### cDNA and double stranded DNA synthesis

Double stranded (ds) DNA was synthesized from the extracted RNA using either FR26RV-N6, FR26RV-Endoh, random hexanucleotides or non-ribosomal \hexanucleotides primer. Firstly, 10 μl of extracted RNA was mixed with 0.1 μM of the primer and 0.5nM of dNTPs (Roche Diagnostics GmbH, Mannheim, Germany). The mixture was incubated at 65 °C for 5 min, and was then immediately chilled on ice for 1 min. Secondly, 7 μl of a reaction mix containing 200U of Super Script III, 40 U of RNase OUT, 0.1 M DTT and 1X first strand buffer (Invitrogen, Carlsbad, CA, USA) was added into the first reaction mixture. The reaction was continued at 25 °C for 10 min, 37 °C for 1 min and 94 °C for 2 min, and then immediately chilled on ice for 2 min. Next, 5U of exo-Klenow fragment (Ambion) and 10U of Ribonuclease H (Ambion) were added into the reaction mixture, which was finally subjected to a double-stranded (ds) DNA synthesis step consisting of 25 °C for 5 min, 37 °C for 1 h and 75 °C for 10 min.

### Random amplification

The resulting dsDNA products generated by FR26RV-N6 and FR26RV-Endoh primers were amplified using FR20RV primer (5′-GCCGGAGCTCTGCAGATATC-3′). PCR amplification was carried out in a total reaction volume of 50 μl consisting of 3 μl of dsDNA, 0.4 μM of primer FR20RV and 45 μl of Platinum PCR supermix high fidelity (Invitrogen). The thermal cycling condition consisted of 94 °C for 2 min and followed by 40 cycles of 94 °C for 30s, 55 °C for 30s and 72 °C for 3 min and 1 cycle of 72 °C for 2 min.

### Next generation sequencing library preparation and sequencing

The resulting dsDNA generated by hexanucleotides or non-ribosomal hexanucleotides and rPCR products were purified with use of QIAquick PCR purification kit (QIAgen GmbH, Hilden, Germany). DNA concentration of the purified products was measured by Qubit dsDNA HS kit (Invitrogen). One nanogram of the purified DNA was then subjected to library preparation steps by using Nextera XT DNA library preparation kit (Illumina, San Diego, CA, USA), according to manufacturer’s instructions. Prior to sequencing, the quantity of the prepared library was measured by using KAPA Library Quant Kit (Kapa Biosystems, Wilmington, MA, USA), following manufacturer’s instructions.

The prepared library was sequenced using MiSeq reagent kit V2 in an Illumina Miseq platform (Illumina). For each run, tested samples were multiplexed and differentiated by double indexes using Nextera XT Index Kit (Illumina).

### Sequence analysis

The sequences generated by Illumina Miseq were analyzed using Geneious 8.1.5 (Biomatters, San Francisco, CA, USA). The obtained sequences were processed to remove primer sequences. Sequence assembly was carried out by using a reference-based mapping strategy available in Geneious (CV-A10, HQ728262; CV-A6, JN582001; CV-A16, JX481738; EV-A71 B5, DQ341363; EV-A71 C4, AB550338), followed by manual editing of the obtained consensus.

Representatives of viral protein 1 (VP1) sequences of CV-A16 (*n* = 39), A6 (38), A10 (29) and EV-A71 (36) of different subgenotypes and from various localities worldwide were used for phylogenetic inference. Pairwise alignment was performed using Geneious alignment tool. Phylogenetic reconstructions were performed using maximum likelihood method (ML) with general time reversible (GTR) nucleotide substitution model available in Geneious package, and support for individual nodes was assessed using a bootstrap procedure (1000 replicates).

The sequences obtained in this study were submitted to NCBI (GenBank) and assigned accession numbers KX430795-KX430824.

## Results

### Non-ribosomal rPCR vs. conventional rPCR

To test whether our modified rPCR, which we named non-ribosomal rPCR, can selectively amplify viral sequences in clinical specimen as compared to the conventional rPCR, two EV-A71 positive swabs with Cp values of 26 (ID.13) and 30 (ID.14) (i.e. high and low viral load) were selected and subjected to random amplification procedures utilizing either FR26RV-N6 or FR26RV-Endoh, and followed by Illumina Miseq sequencing. The total- and percentage of EV-A71 reads, genome coverage and sequencing depth/coverage (i.e. the number of times a single nucleotide was sequenced) were taken into account for comparison.

**Fig. 2:**
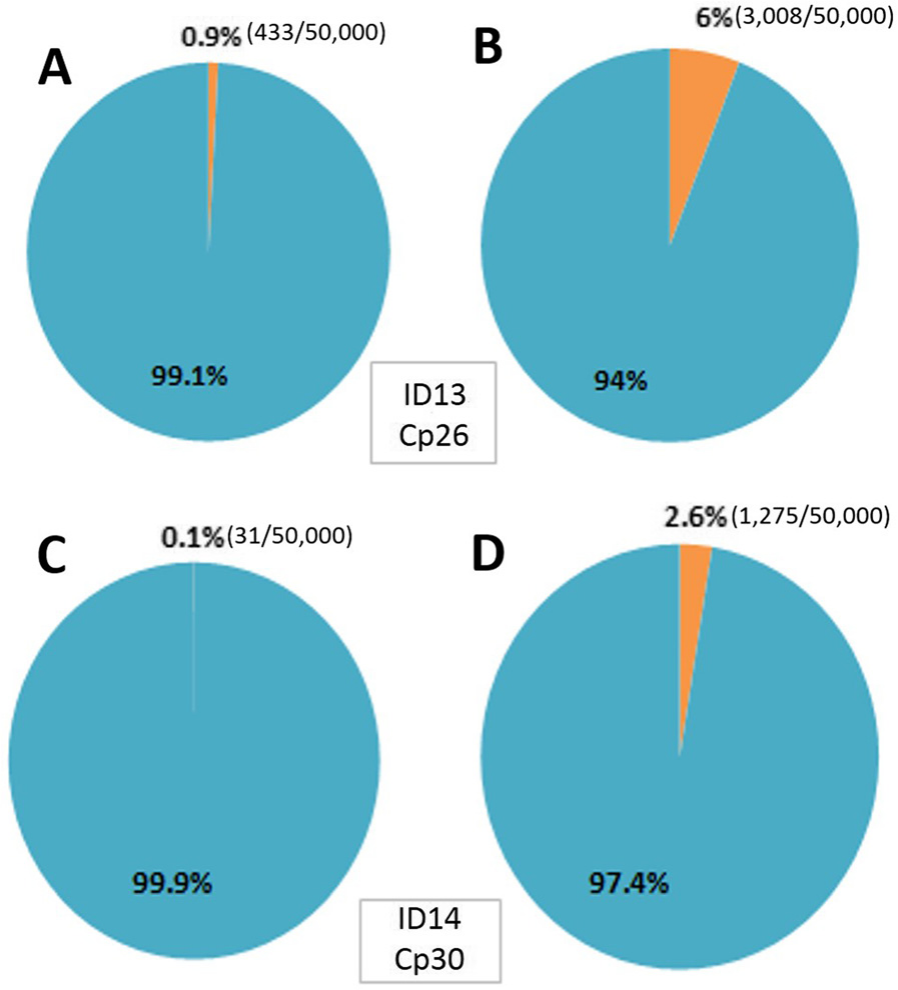
Percentages of EV-A71 reads (in orange) generated by conventional rPCR (**a** for sample ID13 (Cp value: 26) and **c**; ID14 (Cp value: 30)) and by non-ribosomal rPCR (**b**; ID13 (Cp value: 26) and **d**; ID14 (Cp value: 30))

**Fig. 3:**
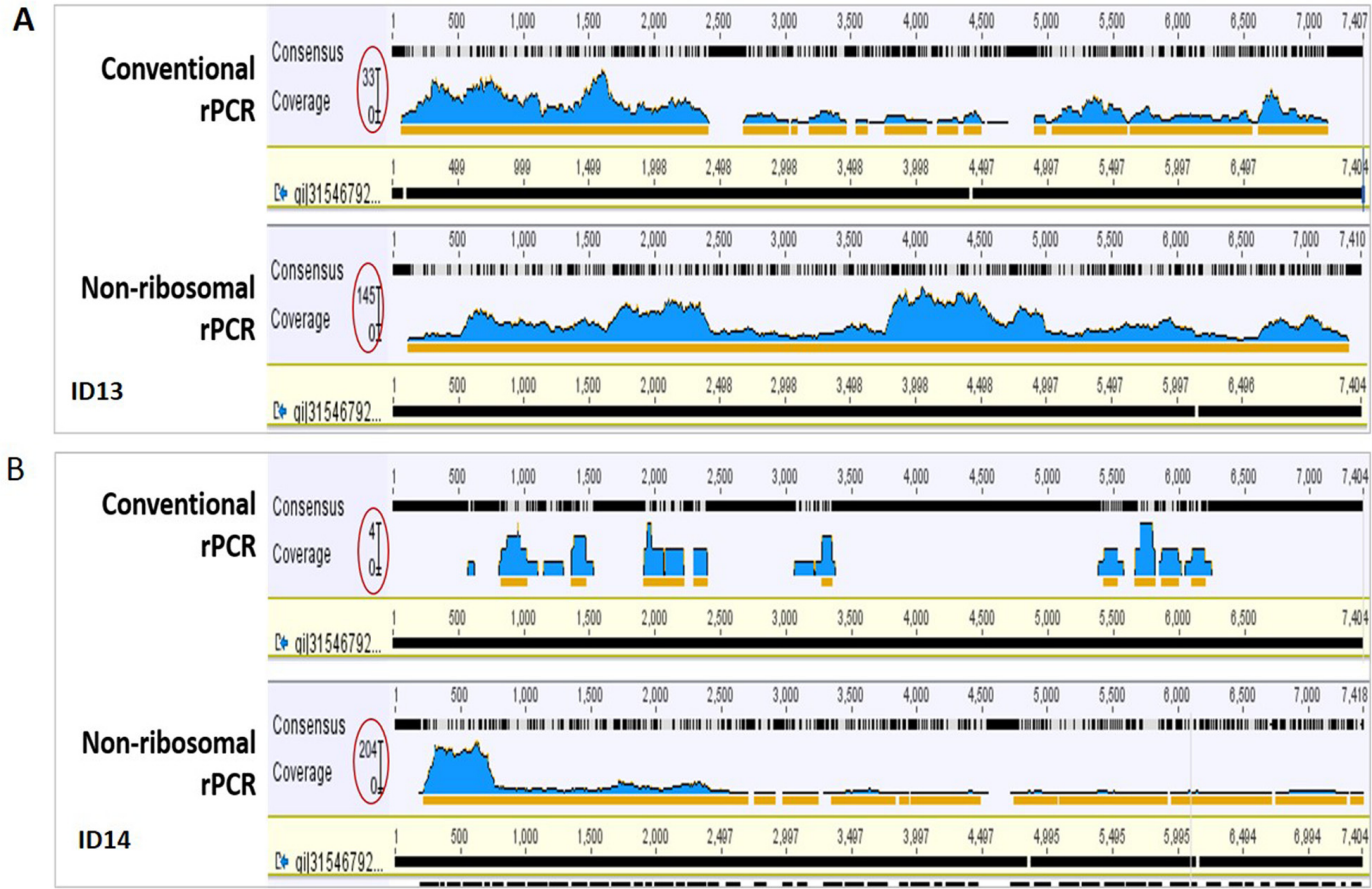
Screen snapshots showing coverage of mapping EV-A71 reads to reference genome, **a** for sample ID13 with a Cp value of 26; non-ribosomal rPCR (lower panel) vs. conventional rPCR (upper panel) and **b** sample ID14 with a Cp values of 30. The genome coverage/sequencing depth is indicated by the Y axis and covered by red circles, and orange lines highlight the sequencing depth of 2 or more

In order to avoid the potential biases introduced by variable number of reads between barcodes, a total of 50,000 reads were randomly taken from each index for the analysis. In both tested EV-A71 positive samples, the total number of EV-A71 reads and the percentage of EV-A71 reads generated by non-ribosomal rPCR based assay was higher than the corresponding outputs generated by the conventional rPCR-based assay; 2.6 % (1275/50,000 reads) vs. 0.1 % (31/50,000 reads) for the sample ID14 with Cp value of 30 and 6 % (3008/50,000 reads) vs. 0.9 % (433/50,000 reads) for the sample ID13 with Cp value of 26 (Fig. 2). Additionally, a higher EV-A71 genome coverage and sequencing depth were also observed in both samples sequenced by non-ribosomal rPCR-based assay (Fig. 3). Taken together, the data indicated that our non-ribosomal rPCR is more viral specific and efficient than the conventional rPCR.

### Non-ribosomal rPCR vs. direct sequencing

Previous studies shown that viral load enrichment by random amplification step resulted in biases in genome coverage [15, 16]. We therefore further evaluated our non-ribosomal rPCR by comparing its performance against that of direct sequencing of dsDNA library generated by hexanucleotide or non-ribosomal hexanucleotide primers. An EV-A71 positive throat swab (sample ID15) with a Cp value of 31 was used. After normalization, the obtained reads of each DNA library were map to an EV-A71 genome (DQ341363.1). Despite biases in terms of sequencing depth across the genome, non-ribosomal rPCR based workflow could generate nearly complete EV-A71 genome sequence (KX430823), while dsDNA library produced by hexanucleotide and non-ribosomal hexanucleotide primers could not (Additional file 1: Figure S1).

### Detection and sequencing of HFMD pathogens: assessment of assay sensitivity and reproducibility

To further evaluate the performance of our non-ribosomal rPCR assay in terms of sensitivity and reproducibility a series of 12 swabs that were EVs real time PCR positive with different common HFMD pathogens (including CV-A6, CV-A10, CV-A16 and EV-A71) and with a wide range of Cp values from 20.8 to 33.3 [12] (i.e. from high to low viral load) (described in Methods section) were included for testing (Table 1). The included samples were tested in duplicate from sample pretreatment to nucleic acid isolation, random amplification by FR26RV-Endoh primers and sequencing by Illumina Miseq, resulting a total of 24 MiSeq datasets (Table 1).

**Table 1:** Result summary of non-ribosomal rPCR and Miseq run

Virus^a^	Sample ID	Sample type	Cp values	% of enteroviral read	% Genome coverage	Internal gap length (bp)	Mean coverage	Accession numbers	Pairwise identity (%)
CV-A6	1	RS	22.69	90.2	99.5	0	26542	KX430795	100
				85.1	100	0	22630	KX430796	
	2	TS	28.34	11.2	97.5	0	1173	KX430797	100
				10.9	95.3	0	1119	KX430798	
	3	RS	30.5	7.9	97.7	0	1822	KX430799	100
				8.3	97.3	57	2244	KX430800	
	4	TS	32.06	7.1	75	1625	1328	KX430801	99.96
				13.3	96.8	41	3061	KX430802	
CV-A10	5	RS	20.92	40.7	99.2	0	11189	KX430803	100
				53.8	98	0	12725	KX430804	
	6	RS	23.59	53.1	97.5	0	17439	KX430805	100
				51.9	97.6	0	14086	KX430806	
	7	RS	26.71	18.2	98	0	4299	KX430807	99.99
				14.5	97	0	3820	KX430808	
	8	TS	33.2	42.4	94	0	12216	KX430809	99.99
				30.5	97.2	26	8412	KX430810	
CV-A16	9	TS	24.97	83.8	99.7	0	3161	KX430811	100
				80.1	99	0	20597	KX430812	
	10	TS	26.72	2	91	71	475	KX430813	100
				2.1	96	0	509	KX430814	
	11	TS	33.26	4.5	96	0	971	KX430815	100
				4.2	86	712	1101	KX430816	
EV-A71	12	TS	31.1	0.2	72.5	1447	5.3	KX430817	100
				0.3	94	0	52.6	KX430818	

^a^Miseq run was multiplexed. Only run output of relevant samples were shown here; CV-A6: coxsackievirus A6, CV-A10: coxsackievirus A10, CV-A16: coxsackievirus A16 and EV-A71 B5: enterovirus A71 subgenogroup B5; TS: Throat swab; RS: rectal swab

#### Assay sensitivity

Illumina Miseq sequencing results showed that in addition to successfully providing correct serotype information (i.e. diagnostic results) in 100 % (24/24) of the tested samples, the assay could generate 17/24 (71 %) genome sequences of HFMD pathogens with the percentages of coverage of between 94 and 100 % (Table 1).

Collectively, of 24 tested samples, whole-genome sequencing success rates of 100 % (8/8), 93 % (13/14) and 71 % (17/24) with genome coverage of 94-100 % without internal gap were achieved among samples with Cp values of ≤25, ≤30 and ≤33.3, respectively (Table 1).

#### Assay reproducibility

To investigate the reproducibility of the assay, we compared the level of sequence identity between the obtained consensuses of the tested sample and its replicate. In 9/12 tested samples the consensuses of both replicates were 100 % identical (Table 1). In the remaining 3 samples, the differences of between 0.01 - 0.04 % were recorded (Additional file 1: Table S2). Additionally, the level of genome coverage, mean coverage (i.e. the numbers of times that a single nucleotide was sequenced) and the percentage of viral reads were comparable between two replicates (Table 1).

### Phylogenetic analysis

Currently there are relatively few whole-genome sequences of CV-A6, CV-A10 and CV-A16 from limited geographical localities available in GenBank. To make more meaningful phylogenetic inference, we therefore first focused our analysis on representative VP1 sequences collected from different geographic locations worldwide.

**Fig. 4:**
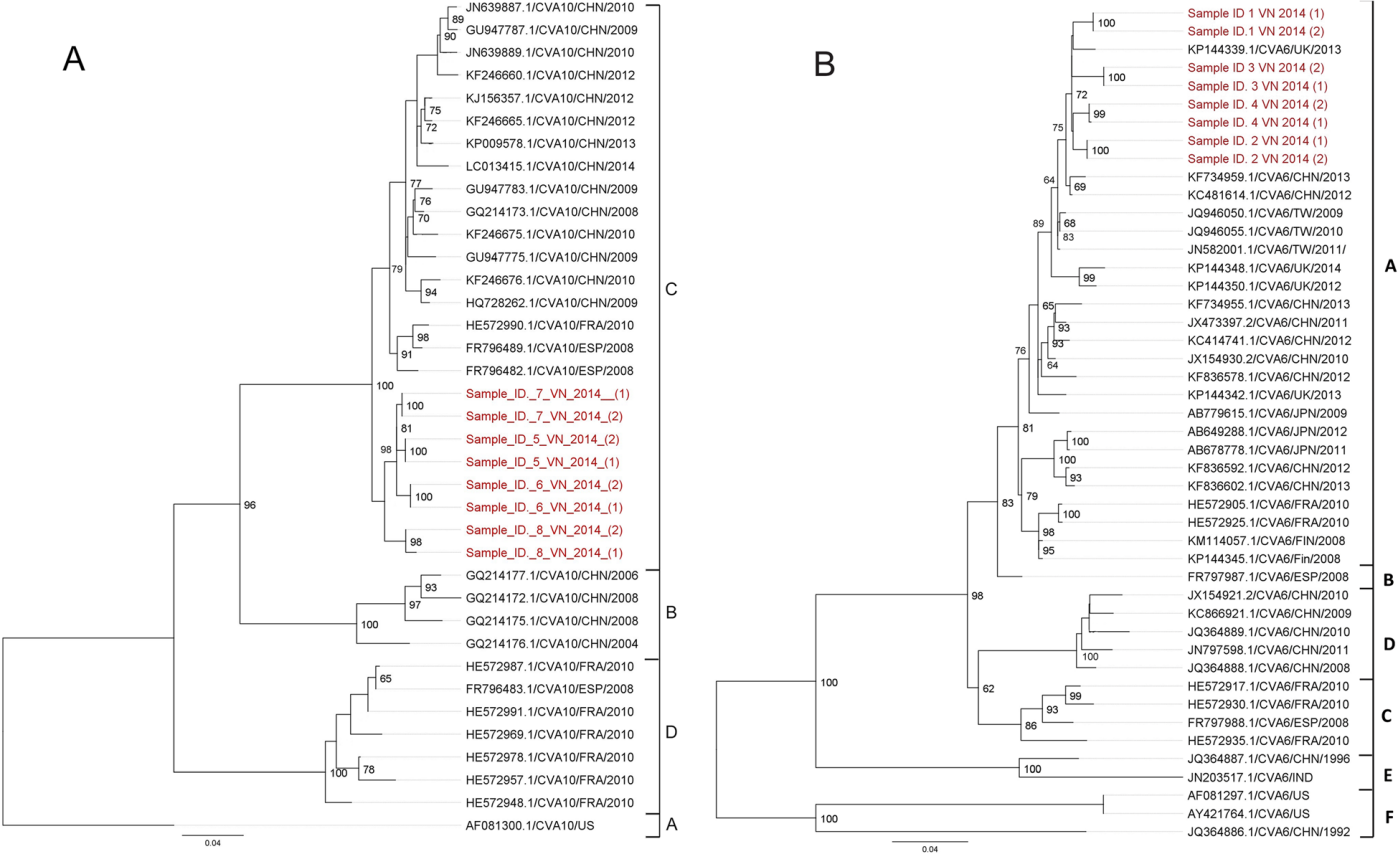
The Maximum likelihood phylogenetic trees based on completed VP1 nucleotide sequences obtained in this study and representatives of VP1 sequences retrieved from GenBank. **a** ML phylogeny of VP1 sequences (894 nt) of CV-A10 strains (*n* = 54); **b** ML phylogeny of VP1 sequences (915 nt) of CV-A6 strains (*n* = 60). Scale bars indicated numbers of nucleotide substitution per site. CHN, China; FRA, France; ESP, Spain; US, United states; IND, India; Fin, Finland; JPN, Japan; TW, Taiwan; UK, United Kingdom; VN, Vietnam

Phylogenetic analysis of VP1 sequences suggested that the EV-A71 strains obtained in the present study sampled in 2012 belonged to subgenogroup C4, whereas the viruses collected in 2013 belonged to subgenogroup B5 (Additional file 1: Figure S1), which reconfirmed our previous finding about the replacement between these two subgenogroups occurring in Vietnam around 2012 [17]. All CV-A16 sequences belonged to genogroup B1a. In Vietnam, this B1a genogroup was first detected in the 2005 outbreak [18] and showed a close relatedness to the viruses circulating in the Asia-Pacific region (e.g. China, Japan, Thailand and Malaysia) (Additional file 1: Figure S2). In contrast, the analysis of CV-A6 sequences indicated that our CV-A6 belonged to genogroup A, which consists of CV-A6 strains sampled from United Kingdom and others viruses from China and Taiwan (Fig. 4b). Likewise, the CV-A10 strains sequenced in the present study belonged to genogroup C consisting of viral trains originating from various parts of the world and associated with HFMD outbreaks in Europe and Asia including in Spain, France and China (Fig. 4a). Similar results in terms of phylogenetic clustering of the sequences were obtained when whole-genome sequences were analyzed separately (data not shown).

## Discussion

Traditionally, obtaining whole-genome sequence of a pathogen requires the design of several overlapping specific PCR primers based on the basis of sequence alignment of the published genome sequences. Although such strategies have been successfully applied for sequencing of HFMD pathogens including EV-A71 and other EV-As [9, 19–21], except for EV-A71, these overlapping primers were designed based on a limited numbers of sequences of EV-As and therefore may not function properly on diverse circulating viral strains whose complete genomes are yet to be sequenced. In addition, to be able to amplify emerging outbreak/novel strain, such viral specific PCR primers often need to be updated regularly, which is always challenging.

There have been several reports regarding the use of random primers, e.g. FR26RV-N6 primer, to generate whole-genome sequence of viral pathogens [22, 23]. However, as FR26RV-N6 primer contains a random hexamer motif at the 3′ end, which is not viral specific, assays may therefore lack specificity when used on materials such as rectal/throat swabs, which contain high amounts of host genetic materials and low concentrations of targeted virus. Meanwhile, Endoh’s non-ribosomal hexanucleotide oligos have recently been successfully used as an alternative to random hexamers for selective amplification of viral RNA in the field of viral pathogen discovery [24–26]. For specific amplification and sequencing of viral pathogens in particular HFMD viruses (which were the focus of the present study) in clinical specimens, we adapted the fixed 5′ end oligo of the normal random PCR and Endoh’s non-ribosomal hexanucleotides to create a novel 96 viral specific rPCR primer set (Additional file 1: Table S1).

When compared back-to-back using EV-A71 positive swabs, our non-ribosomal rPCR evidently outperformed the normal rPCR utilizing FR26RV-N6 primers and direct sequencing of dsDNA libraries generated by either hexanucleotides or non-ribosomal hexanucleotides. In subsequent testing we showed that without the requirement of viral specific PCR, our assay could generate whole-genome sequences of 4 different common HFMD pathogens (including CV-A6, CV-A10, CV-A16 and EV-A71) in either rectal or throat swabs with diverse viral load. Of 24 tested samples with Cp values between 20.9 and 33.2, (nearly) complete genomes were obtained in 17/24 (71 %) samples, representing the first whole-genome sequences of CV-A6, CV-A10 and CV-A16 from Vietnam. In three tested swabs and their replicates, the obtained consensuses occupied between 0.01–0.04 % of differences. This is however below the reported error rate of next generation sequencing (0.1 %). Of note, 2 out of the 4 EV-A71 genomes sequenced in the present study (sample IDs: 13 and 15) were previously recovered (KJ686266 and KX430824) using an overlapping PCRs and deep sequencing based workflow [9, 17]. And pairwise comparisons of the obtained consensuses generated by both workflows revealed only 0.03 % and 0.04 % of variations without amino acid substitution observed (data not shown). Collectively, the data points to the fact that potential biases (if any) introduced by enrichment steps as 40-cycle PCR amplification by FR20RV primer of the present workflow is negligible and that our non-ribosomal rPCR and next-generation based assay is reproducible and sensitive.

Despite the use of non-ribosomal primers and the employment of a sample pretreatment step incorporating centrifugation and DNase treatment to enrich for enteroviral content in the swabs, the percentage of enteroviral reads in the obtained MiSeq libraries ranged between 0.2 and 90.2 %. This might have been attributed to the difference in terms of the compositions of non-enteroviral contents between the samples and/or the viral load of the tested viruses. Meanwhile there have been other reports about alternative sequence-independent whole-genome next-generation sequencing based assays including those incorporating sample pretreatment steps as physical virion enrichment and RNase digestion [27–29]. It is therefore of interest to evaluate the usefulness of those sample pretreatment steps when combined with our non-ribosomal rPCR. Likewise, comparing the performance of our non-ribosomal rPCR with those existing sequence-independent assays warrants further research, which is however beyond the scope of the present study.

For clinical diagnostics, obtaining partial viral genome sequence is sufficient for establishment of the diagnostic result. Exploring the use of next-generation sequencing based assay as a diagnostic tool was an objective in many recent reports [30–32]. In addition, next-generation sequencing has been shown to be able to establish the diagnostics in swabs from HFMD patients that were enterovirus specific PCR negative [33]. Similarly, our assay could sequence and provide correct serotype information of the targeted enteroviruses in all tested samples with Cp values between 20.9 and 33.2, although we did not test our assay on samples with lower viral load (i.e. Cp value of >33.2). Assuming that a Cp value of 33.2 is the assay limit of detection, and a Cp value of <30 is required for the purpose of whole-genome sequencing; among a sample collection from over 1300 HFMD patients enrolled in our ongoing HFMD study in Ho Chi Minh City, Vietnam (data not shown), we can conservatively extrapolate that our assay can detect enterovirus in 97 % and generate complete or nearly complete genome sequence of enteroviruses in 62 % of the RT-PCR positive clinical samples, respectively.

The advantages of random amplification and NGS based assay include: i) there is no requirement for several pathogen specific assays to diagnose diseases caused by multiple pathogens as HFMD, and ii) in addition to providing diagnostic information, the obtained sequencing result is informative for study of viral evolution and identification of the source of an outbreak. Indeed, by analyzing the obtained sequences we were able to reveal interesting insights into the evolution and origin of the CV-A6, CV-A10, CV-A16 and EV-A71 in Vietnam, albeit the sample size was small. As a consequence, further effort to obtain full genome sequences of HFMD causing pathogens is currently ongoing as part of our HFMD research program, which ultimately would lay the foundation for future research focusing on genetic diversity and evolutionary dynamics of HFMD in Vietnam and beyond, and can now be facilitated by our viral specific rPCR and next-generation sequencing based assay.

Our study has some limitations: i) we only evaluated our assay performance on rectal/throat swabs, whereas in HFMD, viral detection in vesicle swab, blood, CSF and urine has been reported, albeit at a lower frequency in the latter 3 sample types. Evaluation of the assay on these sample types is therefore needed, ii) similar to other reports [15, 16], we observed that the level of sequencing depth varied across the genomes of the tested viruses generated in the present study. While in silico investigation did not reveal any biases in terms of binding preference of the non-ribosomal hexanucleotides to specific genomic regions of the targeted viruses, it is attempting to speculate that such biases were attributed to the transposome-based library workflow as previously reported [34], iii) the current high cost (~$USD 75 per sample as compared to $USD 5–8 per one monoplex PCR reaction), low throughput (total operation time is about 5 days to complete) and bioinformatics requirements remain major barriers for next-generation sequencing-based assays to be widely applied in a diagnostic setting, in particular in less developed countries in Asia where HFMD is endemic, iv) the capacity of rPCR and next generation sequencing based assay to detect mixed infection and to identify novel/new viral variants [27, 35, 36] was not explored as it is beyond the scope of this study. For the latter, de novo assembly approach followed by metagenomic analysis using appropriate bioinformatics tool is recommended. Likewise, evaluating the viability of the 96 non-ribosomal hexanucleotides on new viral species discovered from 2005 onward is needed.

## Conclusion

We have successfully developed a non-ribosomal rPCR and next-generation sequencing based assay for sensitive detection and whole-genome sequencing of HFMD pathogens in clinical samples. Our assay can be used to study the genetic diversity and evolutionary biology of HFMD pathogens, which may aid the development of intervention strategies (including vaccines), and guide public health plans in response to future HFMD outbreaks. As next-generation sequencing associated cost has been going down quickly, and once the bioinformatics challenge becomes less burden, one would expect the expanding use of next-generation sequencing based methodologies in clinical research and routine care, both in developed and less developed countries.

## Additional file

Additional file 1: Table S1. List of 96 FR26RV-Endoh and FR20RV primer sequences. **Table S2.** Result summary of consensus sequence variations recorded between 2 replicates of 3 tested swabs. Note: NA: not applicable. **Figure S1.** Screen snapshots showing the mapping results of EV-A71 MiSeq reads to an EV-A71 reference genome of sample ID15; non-ribosomal rPCR assay (bottom panel), non-ribosomal hexanucleotide primers assay (middle panel) and hexanucleotide assay (top panel); the genome sequencing depth is indicated by the Y axis and covered by red circles.** Figure S2.** Maximum likelihood phylogenetic tree based on completed VP1 nucleotide sequences (891 nt) of EV-A71 strains obtained from this study (in bold red) and representatives retrieved from GenBank. Scale bars indicated numbers of nucleotide substitution per site. CHN, China; USA, United states; TW, Taiwan; NL, Netherlands; MY, Malaysia; KOR, Korean; VN, Vietnam. **Figure S3.** Maximum likelihood phylogenetic tree based on completed VP1 nucleotide sequences (891 nt) of CV-A16 strains obtained from this study (in bold red) and representatives retrieved from GenBank. Scale bars indicated numbers of nucleotide substitution per site. CHN, China; US, United states; TL, Thailand; JPN, Japan; AUS, Australia; MY, Malaysia; KOR, Korean; VN, Vietnam. (PDF 783 kb)
