# Supplementary material for: Development and evaluation of a non-ribosomal random PCR and next-generation sequencing based assay for detection and sequencing of hand, foot and mouth disease pathogens
Source: Virol J. 2016 Jul 7;13:125. doi: 10.1186/s12985-016-0580-9 (PMC4937578; doi:10.1186/s12985-016-0580-9)
Supplement: Additional file 1: Table S1. — List of 96 FR26RV-Endoh and FR20RV primer sequences. Table S2. Result summary of consensus sequence variations recorded between 2 replicates of 3 tested swabs. Note: NA: not applicable. Figure S1. Screen snapshots showing the mapping results of EV-A71 MiSeq reads to an EV-A71 reference genome of sample ID15; non-ribosomal rPCR assay (bottom panel), non-ribosomal hexanucleotide primers assay (middle panel) and hexanucleotide assay (top panel); the genome sequencing depth is indicated by the Y axis and covered by red circles. Figure S2. Maximum likelihood phylogenetic tree based on completed VP1 nucleotide sequences (891 nt) of EV-A71 strains obtained from this study (in bold red) and representatives retrieved from GenBank. Scale bars indicated numbers of nucleotide substitution per site. CHN, China; USA, United states; TW, Taiwan; NL, Netherlands; MY, Malaysia; KOR, Korean; VN, Vietnam. Figure S3. Maximum likelihood phylogenetic tree based on completed VP1 nucleotide sequences (891 nt) of CV-A16 strains obtained from this study (in bold red) and representatives retrieved from GenBank. Scale bars indicated numbers of nucleotide substitution per site. CHN, China; US, United states; TL, Thailand; JPN, Japan; AUS, Australia; MY, Malaysia; KOR, Korean; VN, Vietnam. (PDF 783 kb) [file 12985_2016_580_MOESM1_ESM.pdf]

Supplementary Table 1: List of 96 FR26RV-Endoh and FR20RV primer sequences

Primer	Sequence of primer (5'-3')	Primer	Sequence of primer (5'-3')	Primer	Sequence of primer(5'-3')
FR20RV	GCCGGAGCTCTGCAGATATC	33	GCCGGAGCTCTGCAGATATCTAGTCG	66	GCCGGAGCTCTGCAGATATCTAACGC
1	GCCGGAGCTCTGCAGATATCGATATC	34	GCCGGAGCTCTGCAGATATCGTAGAC	67	GCCGGAGCTCTGCAGATATCGGTCAT
2	GCCGGAGCTCTGCAGATATCTAGTAT	35	GCCGGAGCTCTGCAGATATCCTATAG	68	GCCGGAGCTCTGCAGATATCCTCATA
3	GCCGGAGCTCTGCAGATATCTATAGT	36	GCCGGAGCTCTGCAGATATCTAGCTA	69	GCCGGAGCTCTGCAGATATCAATTTG
4	GCCGGAGCTCTGCAGATATCTATATA	37	GCCGGAGCTCTGCAGATATCACTACT	70	GCCGGAGCTCTGCAGATATCCTGGTA
5	GCCGGAGCTCTGCAGATATCATACTA	38	GCCGGAGCTCTGCAGATATCTAACGA	71	GCCGGAGCTCTGCAGATATCTTCATG
6	GCCGGAGCTCTGCAGATATCATATAT	39	GCCGGAGCTCTGCAGATATCCGACTA	72	GCCGGAGCTCTGCAGATATCGCGATA
7	GCCGGAGCTCTGCAGATATCGTGCAC	40	GCCGGAGCTCTGCAGATATCTACTAG	73	GCCGGAGCTCTGCAGATATCACTAAG
8	GCCGGAGCTCTGCAGATATCACTATA	41	GCCGGAGCTCTGCAGATATCAGTAGT	74	GCCGGAGCTCTGCAGATATCGCATAC
9	GCCGGAGCTCTGCAGATATCCGTAAT	42	GCCGGAGCTCTGCAGATATCGTTAAC	75	GCCGGAGCTCTGCAGATATCCAATAT
10	GCCGGAGCTCTGCAGATATCCTATAC	43	GCCGGAGCTCTGCAGATATCGTCTAC	76	GCCGGAGCTCTGCAGATATCACCGTA
11	GCCGGAGCTCTGCAGATATCTATACG	44	GCCGGAGCTCTGCAGATATCTACAAG	77	GCCGGAGCTCTGCAGATATCGTGCTA
12	GCCGGAGCTCTGCAGATATCTATGCG	45	GCCGGAGCTCTGCAGATATCTACCAG	78	GCCGGAGCTCTGCAGATATCACGCTA
13	GCCGGAGCTCTGCAGATATCGATACT	46	GCCGGAGCTCTGCAGATATCTGGATT	79	GCCGGAGCTCTGCAGATATCATGTCG
14	GCCGGAGCTCTGCAGATATCCGTATA	47	GCCGGAGCTCTGCAGATATCTCGTTA	80	GCCGGAGCTCTGCAGATATCAGCTTA
15	GCCGGAGCTCTGCAGATATCGTATAG	48	GCCGGAGCTCTGCAGATATCATAGTA	81	GCCGGAGCTCTGCAGATATCCGACAT
16	GCCGGAGCTCTGCAGATATCCGGTTA	49	GCCGGAGCTCTGCAGATATCATAGTC	82	GCCGGAGCTCTGCAGATATCGCTATA
17	GCCGGAGCTCTGCAGATATCAATAGT	50	GCCGGAGCTCTGCAGATATCCTAGTA	83	GCCGGAGCTCTGCAGATATCGCTATG
18	GCCGGAGCTCTGCAGATATCCGCATA	51	GCCGGAGCTCTGCAGATATCGTACTA	84	GCCGGAGCTCTGCAGATATCTGTAAG
19	GCCGGAGCTCTGCAGATATCATTACG	52	GCCGGAGCTCTGCAGATATCTAAGTT	85	GCCGGAGCTCTGCAGATATCAACTTA
20	GCCGGAGCTCTGCAGATATCTTAACA	53	GCCGGAGCTCTGCAGATATCATATCC	86	GCCGGAGCTCTGCAGATATCATAACG
21	GCCGGAGCTCTGCAGATATCAGTATC	54	GCCGGAGCTCTGCAGATATCTCGATA	87	GCCGGAGCTCTGCAGATATCATGTTA
22	GCCGGAGCTCTGCAGATATCTGTTAA	55	GCCGGAGCTCTGCAGATATCGTACCA	88	GCCGGAGCTCTGCAGATATCTGGTAT
23	GCCGGAGCTCTGCAGATATCACTATT	56	GCCGGAGCTCTGCAGATATCGTATCA	89	GCCGGAGCTCTGCAGATATCTGCGTA
24	GCCGGAGCTCTGCAGATATCTAACCG	57	GCCGGAGCTCTGCAGATATCATACTC	90	GCCGGAGCTCTGCAGATATCGGATAT
25	GCCGGAGCTCTGCAGATATCCGATAT	58	GCCGGAGCTCTGCAGATATCACATTA	91	GCCGGAGCTCTGCAGATATCCATAGC
26	GCCGGAGCTCTGCAGATATCGTATAC	59	GCCGGAGCTCTGCAGATATCATATTG	92	GCCGGAGCTCTGCAGATATCCATACT
27	GCCGGAGCTCTGCAGATATCAATCCA	60	GCCGGAGCTCTGCAGATATCCGTCTA	93	GCCGGAGCTCTGCAGATATCCGGATA
28	GCCGGAGCTCTGCAGATATCTAGCAC	61	GCCGGAGCTCTGCAGATATCCTTAGT	94	GCCGGAGCTCTGCAGATATCTTACTA
29	GCCGGAGCTCTGCAGATATCATATCG	62	GCCGGAGCTCTGCAGATATCCTTACA	95	GCCGGAGCTCTGCAGATATCACTCGT
30	GCCGGAGCTCTGCAGATATCAATATT	63	GCCGGAGCTCTGCAGATATCTTATGC	96	GCCGGAGCTCTGCAGATATCTAAGGT
31	GCCGGAGCTCTGCAGATATCTATAGC	64	GCCGGAGCTCTGCAGATATCATACGC		
32	GCCGGAGCTCTGCAGATATCCTTGTA	65	GCCGGAGCTCTGCAGATATCCGCTTA		

Supplementary Table 2. Summary results of consensus sequence variations recorded between 2 replicates of 3 tested swabs

Virus	Sample ID	Cp value	% pairwise identity	SNP	Location	Depth	Synonymous/ Nonsynonymous
CV-A6	4	32.06	99.96	A→T<br>G→A	5'UTR<br>VP2	1062/3170<br>333/1917	NA<br>Synonymous
CV-A10	7	26.71	99.99	A→G	3D	231/205	Synonymous
	8	33.2	99.99	G→A	2C	205/231	Synonymous

Note: NA: non applicable

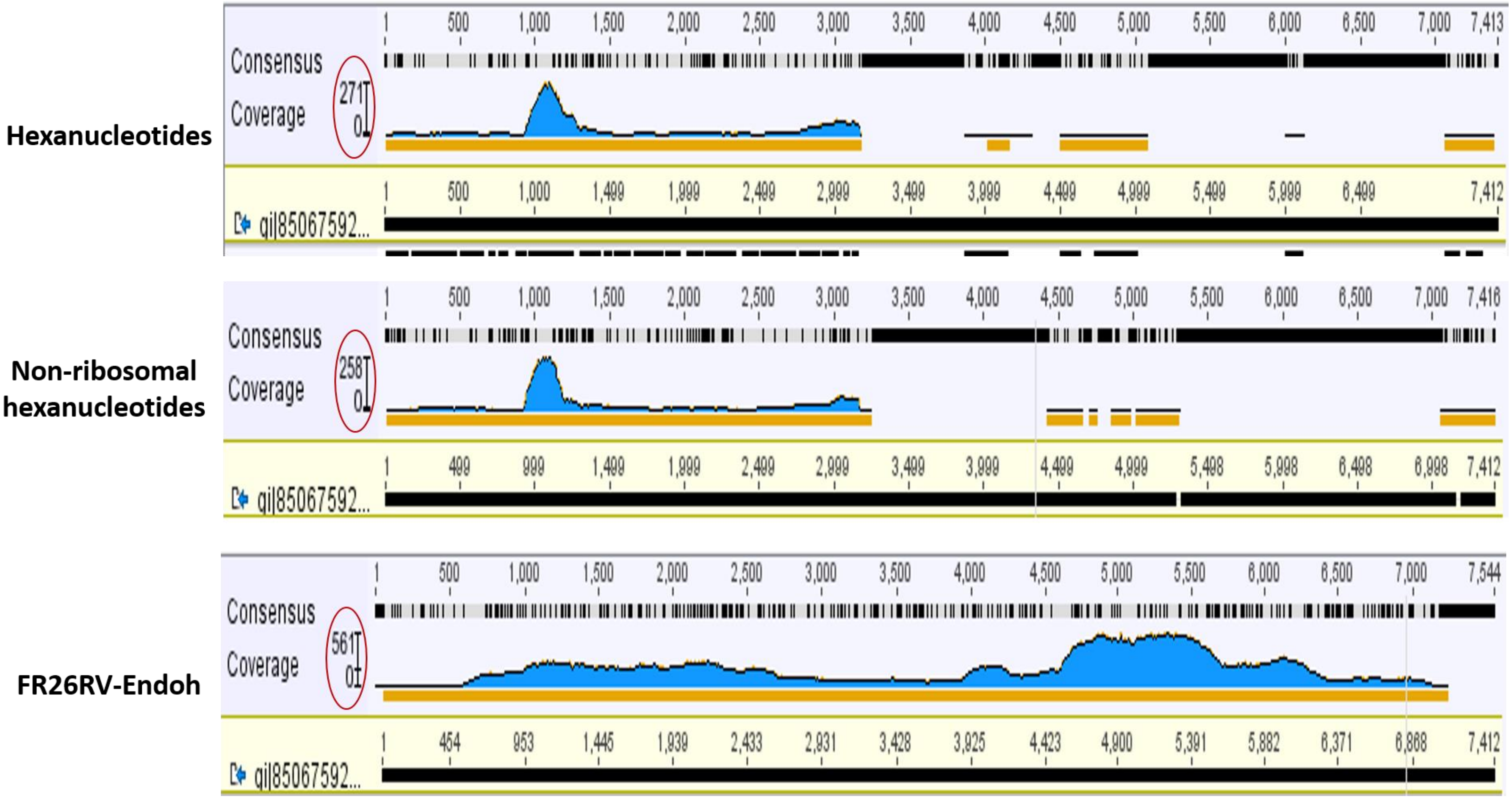

Supplementary Figure 1: Screen snapshots showing mapping results EV-A71 MiSeq reads to an EV-A71 reference genome of sample ID15; non-ribosomal rPCR assay (bottom panel), non-ribosomal hexanucleotide primers assay (middle panel) and hexanucleotide assay (top panel); the genome sequencing depth is indicated by the Y axis and covered by red circles.

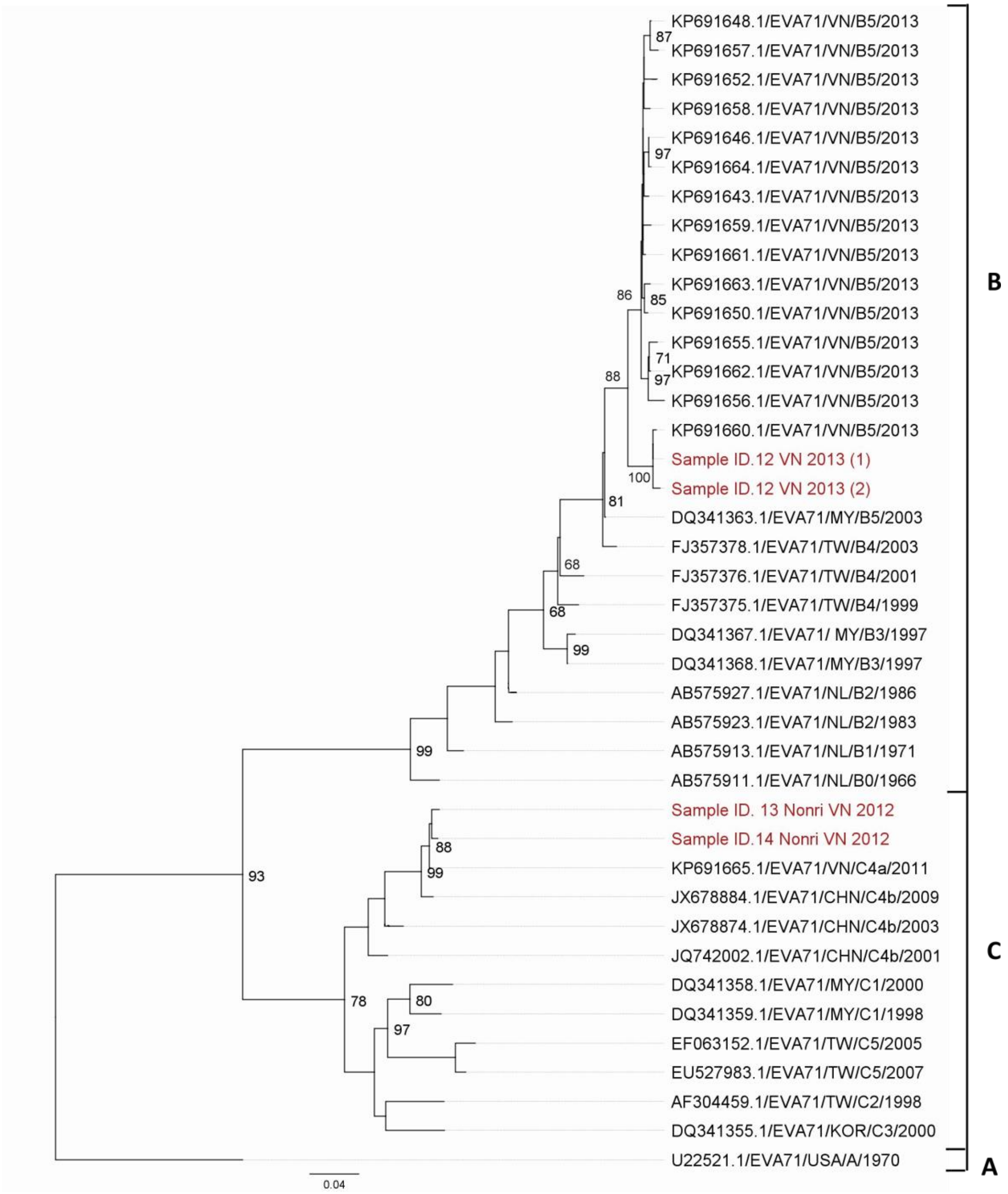

Supplementary Figure 2: Maximum likelihood phylogenetic tree based on completed VP1 nucleotide sequences (891nt) of EV-A71 strains obtained from this study (in bold red) and representatives retrieved from GenBank. Scale bars indicated numbers of nucleotide substitution per site. CHN, China; USA, United states; TW, Taiwan; NL, Netherlands; MY, Malaysia; KOR, Korean; VN, Vietnam.

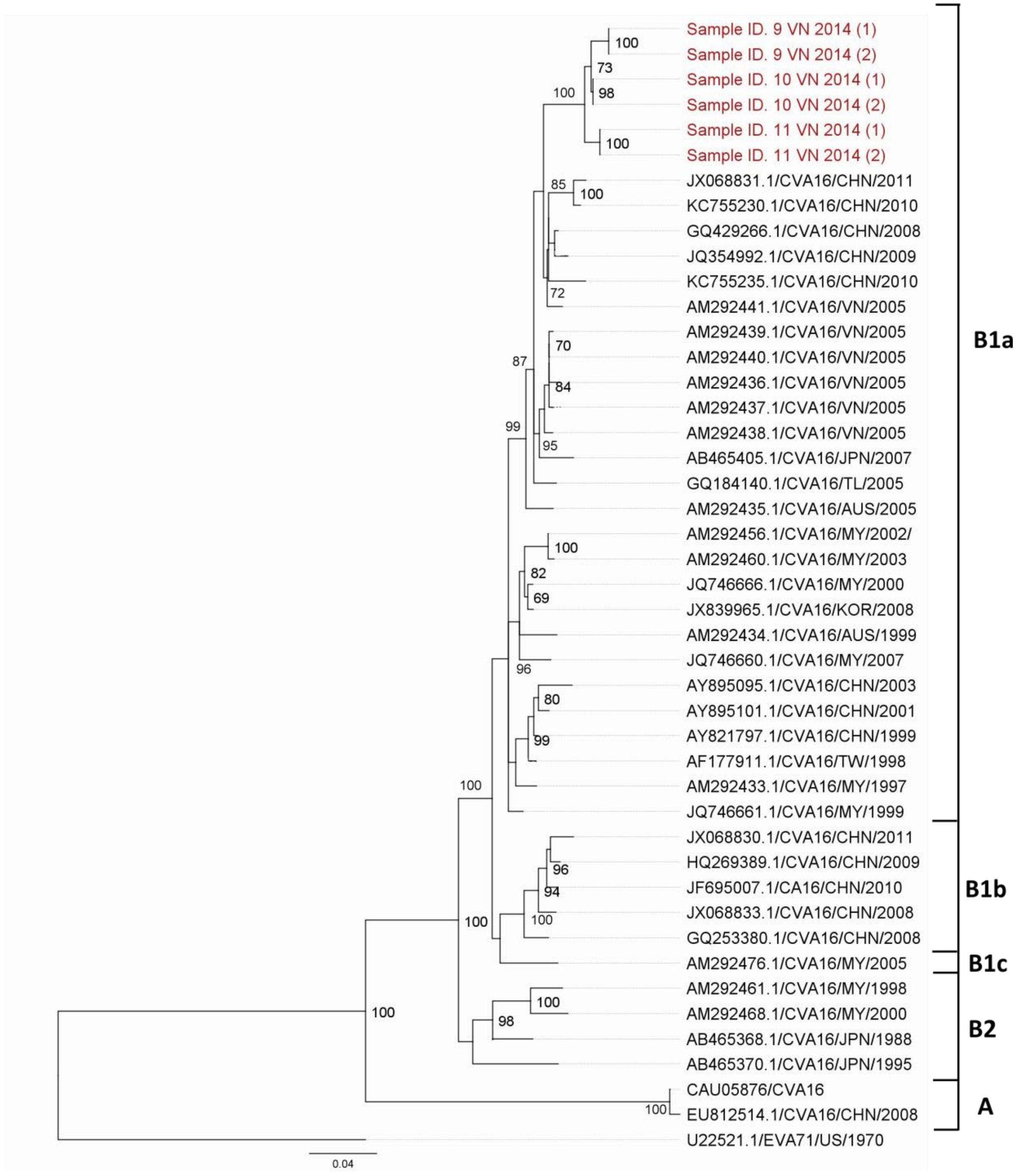

Supplementary Figure 3: Maximum likelihood phylogenetic tree based on completed VP1 nucleotide sequences (891nt) of CV-A16 strains obtained from this study (in bold red) and representatives retrieved from GenBank. Scale bars indicated numbers of nucleotide substitution per site. CHN, China; US, United states; TL, Thailand; JPN, Japan; AUS, Australia; MY, Malaysia; KOR, Korean; VN, Vietnam.
